# Drinking Water Salinity and Maternal Health in Coastal Bangladesh: Implications of Climate Change

**DOI:** 10.1289/ehp.1002804

**Published:** 2011-04-12

**Authors:** Aneire Ehmar Khan, Andrew Ireson, Sari Kovats, Sontosh Kumar Mojumder, Amirul Khusru, Atiq Rahman, Paolo Vineis

**Affiliations:** 1 Medical Research Council/Health Protection Agency Centre for Environment and Health and Department of Epidemiology and Biostatistics, School of Public Health,; 2 Grantham Institute for Climate Change, and; 3 Department of Civil and Environmental Engineering, Imperial College London, London, United Kingdom; 4 Department of Social and Environmental Health Research, London School of Hygiene and Tropical Medicine, London, United Kingdom; 5 Upazilla Health Complex, Dacope, Khulna, Bangladesh; 6 Department of Cardiology, Shaheed Sheikh Abu Naser, Specialised Hospital Khulna, Khulna, Bangladesh; 7 Bangladesh Center for Advanced Studies, Dhaka, Bangladesh

**Keywords:** climate change, hypertension, maternal health, pregnancy, salinity intrusion

## Abstract

Background: Drinking water from natural sources in coastal Bangladesh has become contaminated by varying degrees of salinity due to saltwater intrusion from rising sea levels, cyclone and storm surges, and upstream withdrawal of freshwater.

Objective: Our objective was to estimate salt intake from drinking water sources and examine environmental factors that may explain a seasonal excess of hypertension in pregnancy.

Methods: Water salinity data (1998–2000) for Dacope, in rural coastal Bangladesh, were obtained from the Centre for Environment and Geographic Information System in Bangladesh. Information on drinking water sources, 24-hr urine samples, and blood pressure was obtained from 343 pregnant Dacope women during the dry season (October 2009 through March 2010). The hospital-based prevalence of hypertension in pregnancy was determined for 969 pregnant women (July 2008 through March 2010).

Results: Average estimated sodium intakes from drinking water ranged from 5 to 16 g/day in the dry season, compared with 0.6–1.2 g/day in the rainy season. Average daily sodium excretion in urine was 3.4 g/day (range, 0.4–7.7 g/day). Women who drank shallow tube-well water were more likely to have urine sodium > 100 mmol/day than women who drank rainwater [odds ratio (OR) = 2.05; 95% confidence interval (CI), 1.11–3.80]. The annual hospital prevalence of hypertension in pregnancy was higher in the dry season (OR = 12.2%; 95% CI, 9.5–14.8) than in the rainy season (OR = 5.1%; 95% CI, 2.91–7.26).

Conclusions: The estimated salt intake from drinking water in this population exceeded recommended limits. The problem of saline intrusion into drinking water has multiple causes and is likely to be exacerbated by climate change–induced sea-level rise.

Although scientists do not know with certainty the full extent of the effects of climate change, there is a growing concern that one of the first and most critical impacts will be on the world’s freshwater resources. In coastal Bangladesh, natural drinking water sources, such as rivers and groundwater, are threatened by saltwater intrusion from the Bay of Bengal. According to the Intergovernmental Panel on Climate Change (IPCC), groundwater, crop soils, and many rivers are likely to become increasingly saline from higher tidal waves and storm surges, as a result of climate change impacts (Parry et al. 2007).

The coastal area of Bangladesh is a part of the flat Ganges Delta, which is intersected by large tidal rivers discharging into the Bay of Bengal. The saline front along the 720-km coastline has encroached > 100 km inland into domestic ponds, groundwater supplies, and agricultural land through various estuaries and water inlets, which are interlinked with the major rivers (Allison et al. 2003; Rahman and Bhattacharya 2006). Levels of water salinity have a clear seasonal pattern (Rahman and Ravenscroft 2003) due to rainfall patterns and upstream withdrawal of freshwater (owing to the operation of the Farakka Barrage, which the Indian government uses to regulate flow on the Ganges) during the drier months. Since 1948, river salinity in the southern districts of Patuakhali, Pirojpur, Barguna, Satkhira, Bagerhat, and Khulna has risen by 45% (Integrated Regional Information Networks 2007). Salinity intrusion is likely to increase in the future because of further reduced river flows, increased upstream withdrawal, and longer term climate change–induced decreases in dry season rainfall and sea-level rise.

The coastal population of Bangladesh relies heavily on rivers, tube wells (groundwater), and ponds for washing, bathing, and obtaining drinking water. Domestic ponds, which take up 10% of the total land area (excluding rice paddies), are primarily rain fed but can also mix with saline water from rivers, soil runoff, and shallow groundwater (Rahman and Ravenscroft 2003). Approximately 20 million people living along the coast are affected by varying degrees of salinity in drinking water obtained from various natural sources [Ministry of Environment and Forest (MOEF) 2006].

Guidelines for dietary salt intake have been established by the World Health Organization (WHO), but no guidelines have been released for safe salinity levels in drinking water, except that sodium levels > 0.2 g/L are unacceptable to taste (WHO 2008). High salinity levels in drinking water may have numerous direct and indirect impacts on health. In 2002 the WHO recognized health impacts of consumption of highly saline waters as a priority for investigation under its public health initiatives (WHO 2003). In a survey conducted in 2008, higher rates of (pre)eclampsia and gestational hypertension in pregnant women living in the southwestern coast of Bangladesh, compared with noncoastal pregnant women, were hypothesized to be caused by saline contamination of drinking water (Khan et al. 2008).

In this descriptive study, we estimated salt intake of the population in Dacope, situated in southwestern coastal Bangladesh, and investigated the potential role of salinity in explaining the unusual seasonal pattern of hypertension in pregnancy among the same population.

This study was approved by the Bangladesh Medical Research Council. The research was conducted in accordance with the principles of the Declaration of Helsinki, and it complied with all relevant national, state, and local regulations.

## Materials and Methods

*Study area and populations.* The area included in our study is Dacope Upazilla (subdistrict), situated under the Khulna district in Bangladesh’s southwest coastal region. Dacope is divided into nine administrative unions that comprise 107 villages, with a total population of 157,500 people. It is intersected by a river network, the Passur River being the largest. This region’s rivers are tidal (ranging between 2 and 4.5 m), with semidiurnal, fortnightly, and seasonal variation in water levels.

*Salinity in drinking water: indirect estimates.* The main sources of drinking water in the area include shallow and deep tube wells, ponds, rivers, and rainwater. Water may or may not be filtered before drinking. Rainwater, collected from roofs and stored in large numbers of relatively small containers, is assumed to have negligible levels of salinity.

Indirect estimates of individual salinity intake from groundwater and river water were determined based on salinity data for 1998–2000 from the Centre for Environment and Geographic Information System (CEGIS) in Bangladesh. These data included monthly measurements of salinity in shallow and deep groundwater tube wells at various sites in the Khulna region and in the Passur River. All measurements were converted from decisiemens per meter, a measure of electrical conductivity, into an equivalent concentration of parts per thousand (ppt), assuming that 1 ppt is approximately equivalent to 0.64 dS/m (Ayers and Westcot 1985). We used spatially and temporally (monthly) averaged river and shallow tube-well salinities to estimate average levels of salt consumption from river water and shallow groundwater, respectively. For our estimates, we assumed a conservative water intake of 2 L/day per person.

*Sample of pregnant women and measurement of urinary sodium.* A network of health assistants, under the supervision of the Upazilla Health Complex (UHC) in Dacope, regularly identify and monitor all pregnant women between the ages of 13 and 45 years, in every village of the subdistrict, with a measurement of blood pressure at gestational week 20. Three hundred forty-three pregnant women, a random sample from the monitored women, were enrolled in the study between October 2009 and March 2010 (dry season). The women were sampled in the context of the pilot phase of a larger study that is being planned, where the sampling frame is based on the census. Participants of the study provided written or oral informed consent before participation in the research. The women were all monitored and interviewed at home, using a questionnaire where they indicated their drinking water sources. Direct estimates of salt water intake were made based on salt excretion measured in 24-hr urine samples collected by the women according to verbal instructions provided by research staff. The importance of the completeness of collection was strongly emphasized. Each woman was asked to record the starting time and the finishing time of urine collection. The total volume of urine was recorded, and concentration of sodium was measured in a laboratory at the International Centre for Diarrhoeal Disease Research in Bangladesh.

*Women referred for hypertension in pregnancy.* We also analyzed data from the UHC, the only hospital in the subdistrict. Pregnant women are tested for proteinuria (a symptom of preeclampsia) by health workers during routine pregnancy monitoring at week 20 and if they have high blood pressure or edema. Women diagnosed with mild hypertension with or without preeclampsia are treated at home but are referred to the hospital for more severe or refractory conditions. We determined the prevalence of hypertension in pregnancy among 969 pregnant women (13–45 years of age) who visited the UHC for antenatal care or pregnancy-related complications or were referred to the UHC for severe hypertension between July 2008 and March 2010. Data were collected from medical records on patient diagnosis, age, residence (village), and distance from the UHC. Women were classified as having “hypertension in pregnancy” if they were diagnosed with gestational hypertension (systolic blood pressure > 140 mmHg or diastolic blood pressure > 90 mmHg after the 20th week of pregnancy and not before pregnancy), preeclampsia (high blood pressure in pregnancy, with significant proteinuria), and eclampsia (preeclampsia accompanied by convulsions that could not be attributed to other causes).

*Cutoff point for urinary sodium and blood pressure.* A cutoff point of 100 mmol/day was chosen to classify high salt excretion as an outcome. This threshold was selected based on its association with increased systolic and diastolic blood pressure in the Intersalt study (Elliott et al. 1996; Intersalt Cooperative Research Group 1988). We chose cutoff values for diastolic and systolic blood pressure levels of > 85 mmHg and > 130 mmHg, respectively, because levels higher than those are considered “hypertensive.”

*Statistical analysis.* Using logistic regression, we estimated odds ratios (ORs) and 95% confidence intervals (CIs) for urinary salt excretion > 100 mmol/day according to water source, and for diastolic blood pressure > 85 mmHg and systolic blood pressure > 130 mmHg according to quartiles of urinary sodium concentration. *p*-Values for trend were also estimated by logistic regression. We used a linear regression model to estimate associations between urinary sodium (modeled as a continuous variable) and water sources. We estimated the hospital-based prevalence of hypertension in pregnancy by dividing the number of cases by the total number of pregnant women that visited the UHC and estimated the 95% CI based on normal approximation.

## Results

*Indirect estimates of salt intake from drinking water source and seasonality of salinity levels*. The average level of river salinity in Dacope was 8.21 ppt (range, 1.35–12.9 ppt) during the dry season and 0.64 ppt (0.19–3.90 ppt) during the monsoon season; shallow groundwater salinity averaged 2.6 ppt during the dry season (0.4–11.4 ppt) and 0.60 ppt (0.4–3.8 ppt) during the monsoon season (1998–2000 data from various sites in the Khulna region, obtained from CEGIS) (Table 1). Assuming an average daily water consumption of 2 L, estimated salt intake from drinking water was 5–16 g/day during the dry season (depending on the water source) and 1.2 g/day during the monsoon season.

**Table 1:** Estimated ranges of salt intake in the Dacope Upazilla population (Bangladesh) according to drinking water source and seasonality.

River water							Shallow groundwater						
Salinity*a* (ppt)			Salt intake (g/day)			Salinity*a* (ppt)			Salt intake (g/day)		
Measure	Dry	Monsoon	Dry	Monsoon	Dry	Monsoon	Dry	Monsoon
Minimum		1.35		0.19		2.7		0.38		0.4		0.4		0.8		0.8
Maximum		12.9		3.90		25.8		7.8		11.4		3.8		22.8		7.6
Average		8.21*b*		0.64		16.4		1.28		2.60		0.60		5.2		1.2
**a**Salinity levels for 1998–2000, measured at various sites in Khulna and in the Passur River; obtained from CEGIS, Bangladesh. **b**Equivalent to salt intake of 16.4 g/day (8,210 mg/L × 2 L/day = 16.4 g/day).																

*Results of 24-hr urine analysis of a random sample of 343 pregnant women.* The mean level of sodium in urine was 3.4 g/day (170 mmol/day), with the highest value being 7.7 g/day (387 mmol/day) (Table 2). Women who drank shallow tube-well water had the highest levels of urinary sodium excretion. The OR for urinary sodium levels ≥ 100 mmol/day was 2.05 (95% CI, 1.11–3.80) for women who drank shallow tube-well water versus rain or filtered water (Table 3) (for the choice of the cutoff point, see “Discussion”). Women who drank tube-well water had significantly higher average urinary sodium levels than did women who drank rainwater (β = 35.2; SE = 11.05; 95% CI, 13.5–56.9; *p* = 0.002). In addition; we observed increasing but nonsignificant associations between quartiles of urinary sodium and systolic blood pressure > 130 mmHg [urinary sodium 105.15–155 mmol/day: OR = 1.54 (95% CI, 0.37–6.93); 155.886–204.48 mmol/day: OR = 1.42 (95% CI, 0.34–5.87); > 204.48 mmol/day: OR = 2.41 (95% CI, 0.64–9.13); vs. urinary sodium < 105.15 mmol/day (*p*-value for trend = 0.53)]. Urinary sodium was not associated with diastolic blood pressure > 85 mmHg (data not shown).

**Table 2:** Distribution of urinary sodium excretion by main drinking water source in a random sample of 343 pregnant women in the Dacope Upazilla (Khulna, Bangladesh) [*n*(%)].

Urinary sodium (mmol/day)*a*	Rain + filtered water (*n* = 72)	Pond + river water (*n* = 168)	Shallow groundwater (tube well; *n* = 103)
< 105.14		17 (25.8)		37 (56.1)		12 (18.2)
105.15–155.88		21 (23.9)		45 (51.1)		22 (25)
155.89–204.48		19 (20.4)		46 (49.5)		28 (30.1)
> 204.49		15 (15.6)		40 (41.7)		41 (42.7)
**a**Mean = 169.568 mmol/day; SD = 71.799 mmol/day; range, 19.03–386.67 mmol/day.						

**Table 3:** Urinary sodium above and below 100 mmol/day by main drinking water source in a random sample of 343 pregnant women in the Dacope Upazilla (Khulna, Bangladesh).

Urinary sodium (mmol/day)	Rain + filtered water	Pond + river water	Shallow groundwater (tube well)
< 100		13		32		10
≥ 100		59		136		93
OR (95% CI)		1.0 (reference)		0.94 (0.54–1.62)		2.05 (1.11–3.80)

*Hospital-based prevalence of hypertension.* We identified 90 cases of hypertension in pregnancy among the 969 pregnant women referred to the UHC for problems related to pregnancy. The annual hospital-based prevalence of hypertension in pregnancy was 9.28% (95% CI, 7.46–11.12) (Table 4). We observed a distinct seasonal pattern in prevalence, with a sharp rise in the number of cases diagnosed in pregnant women during the dry season (70 of 576 total pregnancies, vs. 20 of 393 pregnancies in the monsoon season: prevalence OR = 2.39; 95% CI, 1.43–3.99).

**Table 4:** Prevalence rates of hypertension (with or without proteinuria) among pregnant patients 13–45 years of age, recorded between July 2008 and March 2010 in UHC, Dacope, Bangladesh.

Month	No. of cases*a*	Total no. of pregnancies*b*	Prevalence (95% CI)*c*
May–Sept		20		393		5.09 (2.91–7.26)
Oct–April		70		576		12.2 (9.48–14.8)
Total		90		969		9.28 (7.46–11.1)
Prevalence OR = 2.39 (95% CI, 1.43–3.99). **a**With or without proteinuria. **b**Total number of registered patients seeking antenatal care at UHC, Dacope. **c**CIs were calculated using the normal approximation.						

## Discussion

The current recommended dietary intake of sodium is 2 g/day (< 85 mmol/day), according to the report of a joint expert consultation of the WHO and the Food and Agriculture Organization (FAO) in 2002 (Nishida et al. 2004). However, the population of Dacope, Bangladesh, may be consuming 5–16 g/day sodium in drinking water alone during the dry season, depending on their drinking water source. This level of consumption is unacceptable by current standards. Observational studies and clinical trials performed in general populations provide overwhelming evidence that higher salt intake is associated with raised blood pressure (Alderman 2000; He and MacGregor 2007; Law et al. 1991; Midgley et al. 1996). For example, the Intersalt study demonstrated an association between salt intake and elevated blood pressure (Intersalt Cooperative Research Group 1988) and reported that sodium intake > 1.8 g/day (~ 100 mmol/day) was associated with a 3-mmHg increase in systolic blood pressure and 0.1-mmHg increase in diastolic blood pressure (Elliott et al. 1996). To make these data more easily applicable to our population, we used body-mass-index–adjusted conservative estimates from Intersalt. Assuming that these estimates apply to our study population, consumption from 5.2 to 8.8 g/day, on average, could increase systolic blood pressure by approximately 9–18 mmHg to 15–30 mmHg, respectively, potentially leading to a large number of people becoming hypertensive.

Our indirect estimates based on salt levels in rivers and ponds are supported by direct measures of salt excretion in 24-hr urine samples of a random sample of pregnant women in the area. The mean level of daily sodium excretion was 3.4 g/day (170 mmol/day), which is well above the WHO/FAO-recommended levels and above that of many other countries (Brown et al. 2009). Urinary excretion is a reasonable estimate of salt intake in physiological conditions (absorption from food is ~ 98%, whereas urinary excretion is 86% of intake) (Holbrook et al. 1984). As stated above, epidemiologic, experimental, and intervention data support a threshold of 100 mmol/day for the harmful effects of sodium to be expressed (Alderman 2000; Elliott et al. 1996; Midgley et al. 1996). The virtual absence of either hypertension or of a progressive rise in blood pressure with advancing age in populations with an average sodium ingestion < 100 mmol/day, both in the Intersalt study (Stamler et al. 1996) and in other population studies (Page et al. 1981; Poulter et al. 1990), supports the concept of a threshold.

The average hospital-based prevalence of hypertension in pregnancy over the period of the survey was 9.28%. We found a seasonal pattern with an increase in hypertension among pregnant women between November and April, which coincides with the seasonal patterns of low rainfall and upstream flow. We hypothesize that the coastal communities cannot obtain sufficient drinking water from rainwater harvesting during the winter and must therefore use groundwater and ponds as drinking water sources during these months.

The relevance of salt has thus far been considered in the context of diet, and studies assessing the health effects resulting from intake through water have been few and have shown conflicting results. One prospective study in Massachusetts looked at two matched cohorts of high school students from towns with a “high” (272 mg/L) and “low” (20 mg/L) sodium levels in public drinking water and reported that systolic and diastolic blood pressures in the high-sodium region were 3–5 mmHg higher after controlling for dietary salt intake (Calabrese and Tuthill 1981). A subsequent study on high school students conducted in Chicago, Illinois, indicated that diastolic blood pressure was 2 mmHg higher (*p* = 0.040 for males and *p* = 0.016 for females), on average, in the group with 405 mg/L versus 4 mg/L sodium in their drinking water (Hallenbeck et al. 1981). Concerns about elevated rates of hypertension in an Arizona population with water salt levels of 440 mg/L prompted a study of > 700 area residents that showed no difference in the prevalence of hypertension between those who consumed high- versus low-sodium drinking water (Welty et al. 1986). Previous studies assessing the effects of dietary salt intake during pregnancy on the risk of developing preeclampsia and its complications provide insufficient evidence that altering salt intake during pregnancy has any beneficial effect for the prevention of preeclampsia or any other outcome (Duley et al. 2005). However, what we describe are exceptional circumstances where the coastal population is consuming salt levels that are much higher than those consumed by most Western populations.

*Strengths and weaknesses of the study.* We based the study of urinary sodium excretion on a random sample of all pregnant women in the area. Pregnant women are monitored in the Dacope Upazilla, and those that present with complications are referred to the UHC, the only hospital in the area. Therefore, we assume selection bias to be limited.

We observed fewer pregnant women seeking antenatal care at the UHC during the rainy season. One possible explanation for this is that it is difficult for pregnant women to travel because of heavy rains and disruption of the roads, which might bias the prevalence of hospital-based hypertension in pregnancy. However, as mentioned above, we monitor all pregnancies in every household of Dacope, and the health workers refer pregnant women with high blood pressure to the UHC and provide some incentives (transportation, food, and treatment cost). This is likely to reduce bias substantially.

The risk factors for gestational hypertension and (pre)eclampsia (including an immunological hypothesis) are very poorly understood. Although it is unlikely that immunological or other suspected risk factors have a seasonal variation in Bangladesh, we cannot rule out confounding completely.

The analyses did not account for intake of salt from food, a major source, and our data are therefore an underestimation of intake. Our estimates of salt intake from water sources were indirect but were conservative and are supported by the analysis of urinary sodium excretion in the random sample of normal pregnant women in the study. Although measurements may be biased by incomplete urine collection, this would lead to conservative estimates of intake.

Our estimates of river and groundwater salinity levels were based on data for 1998–2000, because we did not have access to more recent data. As both government and nongovernmental organization reports have shown that salinity in rivers and groundwater increases annually [Institute of Water Modeling (IWM) 2003; MOEF 2006], these values would lead to conservative estimates of salinity levels and therefore an underestimation of salt intake by the Dacope population.

As a further limitation, for convenience we divided women into “hypertensive” and “not hypertensive” groups, although it is known that the relation between blood pressure and disease outcomes is continuous with no clear evidence of thresholds.

*Causes of rising salinity.* Salinity in surface and groundwater is determined by a complex combination of factors, including river flow, tides, precipitation, estuarine circulation, water and land management practices, and also sea-level rise and other climatic variables. Salinity in Bangladesh river networks is reasonably well understood, with empirical (Aerts et al. 2000) and physically based (Alam 2003; IWM 2003; MOEF 2006; UK Department for Environment, Food and Rural Affairs 2007) models having been successfully applied to simulate salinity levels. The reduction in upstream freshwater flow from the Ganges, which has dropped off significantly in the Padma (Bangladesh branch of the Ganges River) since India’s commission in 1975 of the Farakka Barrage (Mirza and Sarker 2004), has increased salinity levels in river waters near the coast. The Padma flows at less than a quarter of its capacity during the dry season (October–April), and water flow in the downstream network of rivers, which often stops altogether, is insufficient to wash tidal water back out to sea (Mirza and Sarker 2004). This man-made situation may be the main driver for the wider problem, but the mechanisms by which the water supplies—shallow groundwater and the numerous ponds—develop increasing levels of salinity are not completely understood.

Sea-level rise is likely to play a significant role in increasing salinity in natural drinking water sources in the future. The distance of the salinity intrusion inland, as well as the extent of salinity in the coastal areas, is expected to increase with rising sea levels (MOEF 2006). Estimates of the amount of sea-level rise over the coming century range from 0.2 to 0.6 m (Parry et al. 2007). The Fourth Assessment Report of the IPCC states with “high confidence” that marine and coastal ecosystems in South and Southeast Asia will be affected by sea-level rise as a consequence of climate change (Parry et al. 2007), a critical factor that makes Bangladesh especially vulnerable (MOEF 2006).

## Conclusions

Discovery of the presence of high levels of salt in drinking water sources in rural coastal Bangladesh is a cause of public health concern and a challenge for the government of Bangladesh, donor communities, and nongovernmental organizations. Our findings suggest that the mean sodium intake in pregnant women is well above WHO/FAO–recommended levels and above those of many other countries. We hypothesize that increasing salt intake during the dry season might contribute to the seasonal pattern of hypertension in pregnancy in coastal Bangladesh, and the problem may be exacerbated by future sea-level rise and environmental change. Hypertension in pregnancy is associated with increased rates of adverse maternal and fetal outcomes, both acute and long term, including impaired liver function, low platelet count, intrauterine growth retardation, preterm birth, and maternal and perinatal deaths (Sibai 2002). The adverse outcomes are substantially increased in women who develop superimposed (pre)eclampsia.

With a growing concern for rising salinity, awareness and interest in climate change impacts on water sources are also increasing. Adaptation practices to improve coping mechanisms and reduce vulnerabilities of communities are being advocated, including measures to increase storage capacity of rainwater and apply desalination processes. More research is required to improve the management of fresh surface water and groundwater resources in these areas and to explore rainwater harvesting as a sustainable solution.

Bangladesh stands at the forefront of saltwater contamination. However, the same trend potentially affects all 11 Asian large river deltas, and other major deltas, notably the Nile and the Mississippi (Parry et al. 2007).
